# Diagnosis of brain tumors using dynamic contrast-enhanced perfusion imaging with a short acquisition time

**DOI:** 10.1186/s40064-015-0861-6

**Published:** 2015-02-24

**Authors:** Takashi Abe, Yoshifumi Mizobuchi, Kohei Nakajima, Yoichi Otomi, Saho Irahara, Yuki Obama, Mungunkhuyag Majigsuren, Delgerdalai Khashbat, Teruyoshi Kageji, Shinji Nagahiro, Masafumi Harada

**Affiliations:** Department of Radiology, Institute of Health Biosciences, The University of Tokushima Graduate School, 3-18-15, Kuramoto-cho, Tokushima City, Tokushima 770-8509 Japan; Departments of Neurosurgery, Institute of Health Biosciences, The University of Tokushima Graduate School, Tokushima, Japan

**Keywords:** Magnetic resonance imaging (MRI), Dynamic contrast enhanced (DCE) perfusion, Brain tumor, Two-compartment model analysis, Pharmacokinetic model analysis

## Abstract

This study sought to determine the diagnostic utility of perfusion parameters derived from dynamic contrast-enhanced (DCE) perfusion MRI with a short acquisition time (approximately 3.5 min) in patients with glioma, brain metastasis, and primary CNS lymphoma (PCNSL).

Twenty-six patients with 29 lesions (4 low-grade glioma, 13 high-grade glioma, 7 metastasis, and 5 PCNSL) underwent DCE-MRI in a 3 T scanner. A ROI was placed on the hotspot of each tumor in maps for volume transfer contrast *K*^*trans*^, extravascular extracellular volume *V*_*e*_, and fractional plasma volume *V*_*p*_. We analyzed differences in parameters between tumors using the Mann–Whitney U test. We calculated sensitivity and specificity using receiver operating characteristics analysis.

Mean *K*^*trans*^ values of LGG, HGG, metastasis and PCNSL were 0.034, 0.31, 0.38, 0.44, respectively. Mean *Ve* values of each tumors was 0.036, 0.57, 0.47, 0.96, and mean *Vp* value of each tumors was 0.070, 0.086, 0.26, 0.17, respectively. Compared with other tumor types, low-grade glioma showed lower *K*^*trans*^ (*P* < 0.01, sensitivity = 88%, specificity = 100%) and lower *V*_*e*_ (*P* < 0.01, sensitivity = 96%, specificity = 100%). PCNSL showed higher *V*_*e*_ (*P* < 0.01, sensitivity = 100%, specificity = 88%), but the other perfusion parameters overlapped with those of different histology.

Kinetic parameters derived from DCE-MRI with short acquisition time provide useful information for the differential diagnosis of brain tumors.

## Introduction

The differential diagnosis of brain tumor is critical to determining optimal therapy and estimating prognosis (DeAngelis 2001). High grade glioma, brain metastasis, and primary central nervous system lymphoma (PCNSL) are common types of brain malignancies in adults and can sometimes present similar results on conventional MR imaging (Ma et al. 2010). Dynamic contrast-enhanced (DCE) imaging, which allows for noninvasive evaluation of tumor vascularity, has been widely used to assess the physiology of brain tumor vascularity (Tofts 1996; Tofts et al. 1999).

Dynamic acquisition of images during contrast enhancement allows for the specific descriptive parameters related to local microvasculature characteristics to be calculated. Both relaxivity (T1)- and susceptibility (T2*)-based approaches have demonstrated good potential for measuring the characteristics of tumor vasculature (Quarles et al. 2012). Methods to assess changes in tissue T1 following contrast agent injection are commonly termed DCE-MRI and have been widely performed to assess microvascular permeability (Tofts 1996; Tofts et al. 1999). In DCE-MRI, the signal intensity change can be measured with sufficient temporal resolution and is related to tissue contrast agent concentration.

The pharmacokinetic (PK) model introduced by Tofts et al. can also be used to calculate volume transfer contrast *K*^*trans*^, volume of extravascular extracellular space *V*_*e*_, and fractional plasma volume *V*_*p*_ (Tofts 1996; Tofts et al. 1999). As such, DCE-MRI can provide information on the blood microcirculation of tumors that cannot be acquired from conventional MRI (Tofts 1996; Tofts et al. 1999; Patankar et al. 2005; Xyda et al. 2012; Sorensen et al. 2009; Bisdas et al. 2011; Mills et al. 2006). In the brain, previous studies have used these kinetic parameters to evaluate glioma grade (Patankar et al. 2005), differential diagnosis (Xyda et al. 2012), treatment effects in primary brain tumors (Sorensen et al. 2009), diagnosing recurrence from radiation injury (Bisdas et al. 2011) and predicting prognosis (Mills et al. 2006).

DCE data measured with sufficient temporal resolution and acquisition time can provide useful results in PK model analysis (Tofts 1996; Larsson et al. 2013). Acquisition times of over 5 min have been used for the diagnosis of brain tumors in recent years (Bisdas et al. 2011; Aref et al. 2008; Awasthi et al. 2012; Bagher-Ebadian et al. 2012; Jia et al. 2012) and are recommended to maintain reliability (Larsson et al. 2013). But due to practical time limitations for an MRI examination, a DCE sequence with a short acquisition time and high diagnostic performance is required. Although DCE sequences with short acquisition times result in overestimated *K*^*trans*^ and underestimated *V*_*e*_ and *V*_*p*_, the resultant error is not so large at acquisition times of 3–4 min. Further, when using the same DCE protocol for different tumor types, parametric errors occur in the same direction, so their resulting distributions could be unchanged and diagnostic utility is preserved. In fact, there have been investigations on brain, head and neck, and breast neoplasms using DCE sequences of less than 5 min and the Tofts model (Awasthi et al. 2012; El Khouli et al. 2011; Shukla-Dave et al. 2012). Additionally, for use in clinical practice, reduced operation time is important and can be achieved with automated post-processing and fixed T1 method (Haacke et al. 2007), which uses preselected T1 value as a precontrast T1 value (T10) of the target organ to reduce the noise derived from T1 map and to reduce total acquisition time.

Against this background, the main purpose of this study was to investigate the diagnostic utility of DCE-MRI in diagnosing glioma, metastasis, and PCNSL tumors using a relatively short acquisition time and to reduce operation time using automated post-processing and a fixed T1 method.

## Materials and methods

This study was approved by the university hospital of Tokushima clinical trial center for developmental therapeutics, and informed consent was obtained from all patients prior to enrollment.

### Patients

Fifty-two consecutive patients who underwent contrast-enhanced MRI for diagnosing suspected brain tumor between December 2012 and December 2013 were eligible for this study. From this group, those with glioma, metastasis, and PCNSL were included in the analysis. Diagnosis was made histologically or clinicoradiologically. Clinicoradiological diagnosis was made by consensus of two experienced neuroradiologists. Metastases were defined as newly emerging nodules in cancer patients; and recurrence of glioma were defined as a steady increase in contrast enhanced T1-weighted images. MRI follow-up was performed at 2-month intervals or sooner. Twenty-six patients (17 men and 9 women; mean age 61 years, age range 35–84,years; 29 lesions) were included in the analysis. The time from examination to diagnosis was recorded. We divided tumors into four groups: low grade glioma (LGG), high grade glioma (HGG), metastasis, and PCNSL. Table 1 summarizes the patient information.

**Table 1:** **A summary of patient information**

	**No. of cases (male)**	**Mean age, years (range)**	**Pathology**
LGG	4 (1)	53.3 (35–77)	3 oligodendrogliomas, 1 diffuse astrocytoma
HGG	13 (9)	59.2 (34–84)	1 anaplastic astrocytoma, 1 anaplastic oligodendroglioma, 1 gliosarcoma, 8 glioblastomas, 2 recurrent high grade glioma
Metastasis	6 (4)	64.3 (48–77)	2 lung cancers*, 2 breast cancers*, 1 gastric cancer, 1 colon cancer*
Primary CNS lymphoma	3 (3)	69.0 (55–78)	3 diffuse large B-cell lymphomas

*:Three cases were diagnosed clinically (1 lung cancer, 1 breast cancer and colon cancer). The others were diagnosed pathologically.

### Imaging protocol

Examinations were performed with a 3 T MR scanner (Discovery 750, GE Healthcare, Milwaukee, WI) using a standard eight-channel head coil. Pre-contrast T1-weighted images, T2-weighted images, diffusion-weighted images, arterial spin labeling images, and MR spectroscopy were acquired. Subsequently, DCE-MRI and post-contrast T1-weighted images were acquired with contrast agent (Gd-DTPA, 0.1 mmol/kg; Magnevist, Bayer HealthCare, Berlin, Germany). We performed 3D T2*-weighted angiography and DTI in selected patients.

DCE-MRI was acquired using a 3D fast spoiled gradient echo sequence with TR = 4.4 ms, TE = 1 ms, flip angle = 12°, field of view = 300 × 210 mm, matrix = 128 × 90, slice thickness = 8 mm, and number of slices = 16, consisting of 64 phases with a temporal spacing of 3.3 s. We chose TR, TE, and flip angle in accordance with those of an ordinary spoiled gradient echo sequence. Total scan time was 3 min and 31 s. A gadolinium-contrast agent was injected with a power injector (Medrad, Indianola, PA) at a rate of 2.5 ml/s after two cycles of dynamic scan. Immediately afterward, 20 ml of saline was injected at the same rate.

### Image analysis

All imaging data were transferred from the scanner to a workstation (Advantage Workstation 4.6, GE Medical Systems, Milwaukee, WI). We analyzed DCE data using the Tofts model implemented in the commercially available software GenIQ (GE Medical Systems, Milwaukee, WI). We used 3-dimensional, rigid motion correction, which conduct a combination of rotational movement and translation in all cases. Referring to the temporal changes of the signal intensity of all voxel, the software automatically extracts the pixels that considered to arteries and veins. In the analysis, we used fixed T1 method (Haacke et al. 2007) and default T10 value (T10 = 1000 ms).

### Data analysis

We selected enhanced lesions with a minor axis of more than twice the slice thickness (16 mm) and include them in the following analysis. In each map, we set an ROI of approximately 100 mm^2^ at the hot spot of the tumor. We measured average *K*^*trans*^, *V*_*e*_, and *V*_*p*_ in each tumor. We then assessed the correlation of PK model parameters with different parametric maps and tumor histology.

### Statistical analysis

We first calculated correlations between the DCE parameters *K*^*trans*^, *V*_*e*_, and *V*_*p*_ using Spearman’s rank correlation coefficient. We then assessed the correlation between DCE parameters and tumor histology. Results are expressed as mean ± standard deviation. Statistical difference between tumors was determined using the Mann–Whitney U test. A *P* value of less than 0.05 was considered statistically significant.

Finally, we assessed the utility of *K*^*trans*^, *V*_*e*_, and *V*_*p*_ in diagnosis of the brain tumors. From the results of this analysis, we selected a group of tumors with distinct PK parameters. We performed receiver operating characteristics (ROC) curve analysis for selected tumors to evaluate the optimal cutoff value, sensitivity, and specificity.

All statistical analysis was performed using Excel Statistics 2012 (Social Survey Research Information Co., Ltd., Tokyo, Japan) with Excel 2010 (Microsoft Co., Redmond, WA).

## Results

The time from examination to diagnosis was 5 days (3 and 12 days: 25^th^ and 75^th^ percentiles). Data transfer and post-processing took approximately 10–12 min.

**Figure 1:**
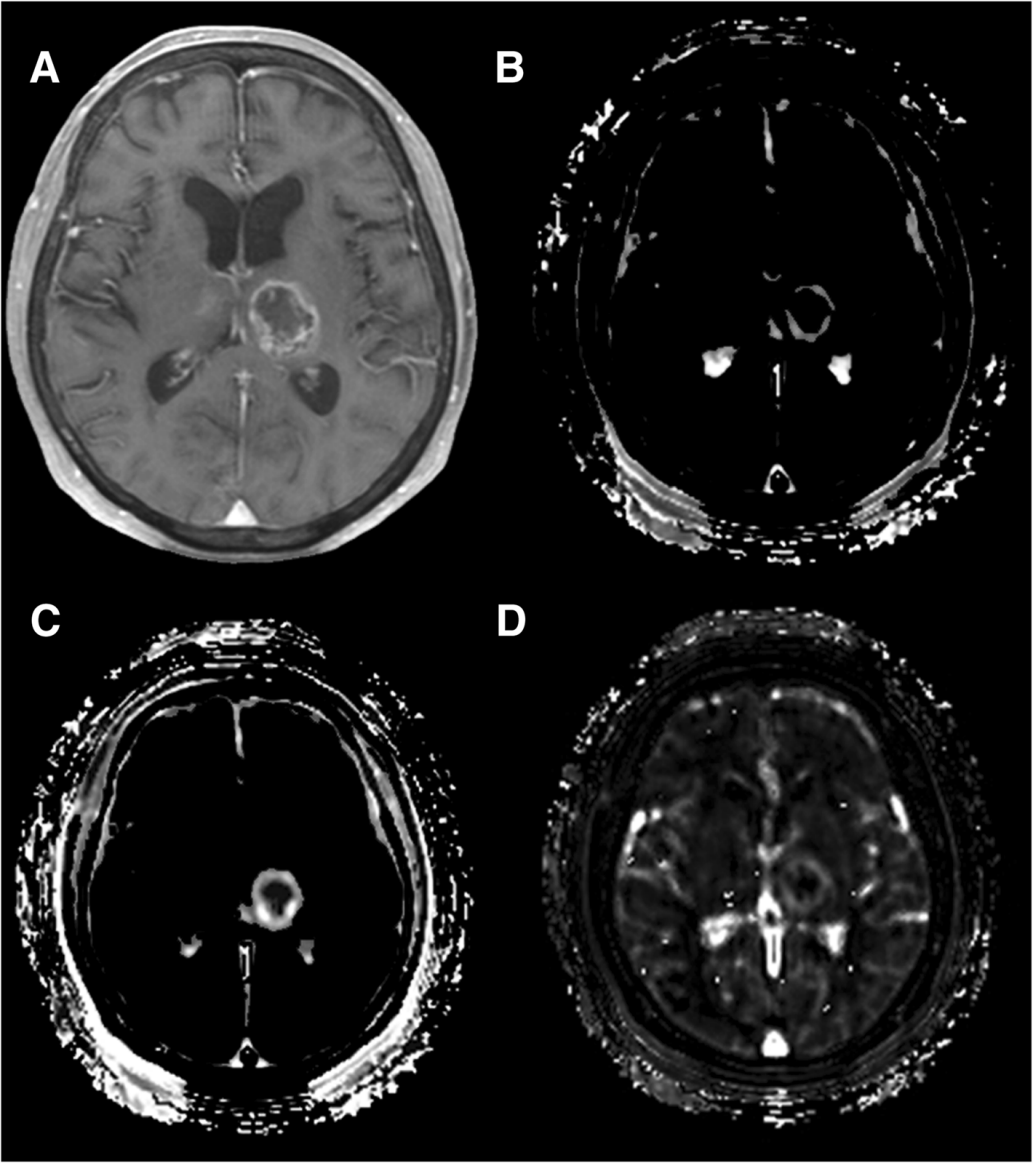
**Magnetic resonance imaging of a glioblastoma. A**, Axial post-contrast T1-weighted image shows a ringed enhanced lesion in the left thalamus and subtle enhancement in the right thalamus. **B**, **C**, and **D**, Three kinetic parametric maps show increased vascular permeability (B; *K* ^*trans*^ map), leakage space (**C**; *V*_*e*_ map), and plasma volume (**D**; *V*_*p*_ map) corresponding to the enhanced area on the contrast-enhanced MRI.

Contrast-enhanced T1-weighted imaging for a representative glioblastoma case (Figure 1) showed increased *K*^*trans*^, *V*_*e*_, and *V*_*p*_. There was an intermediate correlation between *K*^*trans*^ and *V*_*e*_ (*R*^*2*^ = 0.41), while *V*_*p*_ showed relatively weak correlations with *K*^*trans*^ and *V*_*e*_ (*R*^*2*^ = 0.17 and 0.18, respectively)

**Figure 2:**
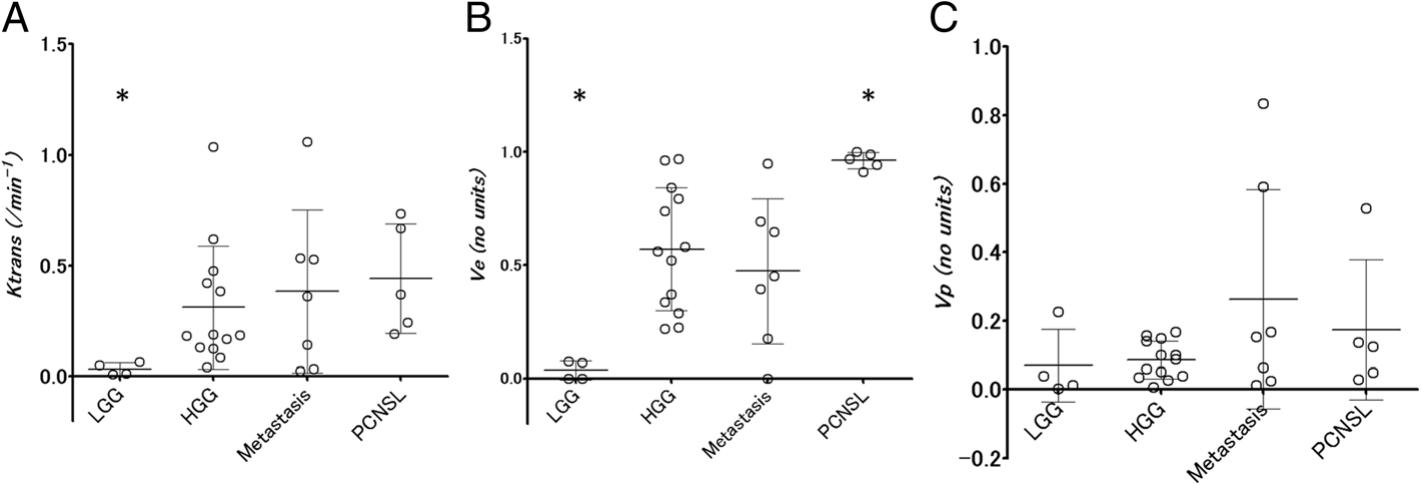
**Scatter plot (mean ± standard deviation) shows 3 kinetic parameters for 4 brain tumor types.** **A**: *K* ^*trans*^, **B**: *V*_*e*_, **C**: Vp. LGG: low grade glioma, HGG: high grade glioma, PCNSL: primary CNS lymphoma, *: significant difference (P < 0.05, Mann–Whitney U test) between tumor groups.

LGG showed lower *K*^*trans*^ and *V*_*e*_ than the other malignant tumor types (*P* < 0.01). Among the other tumor types, *K*^*trans*^ values overlapped. Lymphoma showed extremely high *V*_*e*_ (*P* < 0.01), but *V*_*e*_ for HGG and metastasis overlapped. No statistical differences were found for *V*_*p*_ (Figure 2).

Area under the ROC curve for differentiating LGG and PCNSL was highest for *V*_*e*_ (LGG: 0.97, PCNSL: 0.95). A cutoff value of *K*^*trans*^ = 0.0848 for diagnosis of LGG provided the best good combination of sensitivity and specificity (0.88 and 1.0, respectively). A cutoff value of *V*_*e*_ = 0.18 for diagnosis of LGG provided the best good combination of sensitivity and specificity (0.96 and 1.0, respectively). A cutoff value of *V*_*e*_ = 0.912 for diagnosis of PCNSL provided the best combination of sensitivity and specificity (1.00 and 0.88, respectively).

## Discussion

The results of this study indicate that kinetic parameters acquired from DCE perfusion study with short acquisition time supplement conventional imaging in predicting tumor histology. This method takes only about 15 min (scan time of 3.5 min plus post-processing time of 10–12 min) and is not subject to operator-dependent bias, making it highly feasible in clinical settings.

*K*^*trans*^ and *V*_*e*_ demonstrated modest correlation with each other. *V*_*e*_ was the most useful parameter in diagnosing LGG and PCNSL, while *K*^*trans*^ was effective in differentiating LGG from the other tumors. *V*_*p*_ failed to prove useful in differentiating brain tumor types in this study group.

Although the utility of the two-compartment PK model methods in brain tumor diagnosis has been reported (Patankar et al. 2005; Xyda et al. 2012; Sorensen et al. 2009; Bisdas et al. 2011; Aref et al. 2008; Awasthi et al. 2012; Bagher-Ebadian et al. 2012; Jia et al. 2012), it has not extended to clinical practice. One of the reasons for this is the long acquisition time. In the present study, we confirmed that the diagnostic performance of DCE analysis using a short acquisition time is comparable to that of methods in previous studies. We believe, therefore, that this method provides new and useful performance improvements for tumor diagnosis.

Another method, first-pass pharmacokinetic model (FPPM) analysis, can be performed from DCE analysis data with an ultra-short acquisition time of about 1 min (Li et al. 2000). *K*^*trans*^ and *V*_*p*_ can also be calculated with this method and are comparable to the data obtained from conventional PK model analysis (Harrer et al. 2004). Since, in the FPPM method, tracer concentration in arterial blood plasma is assumed to be much larger than that in the extravascular extracellular component, *V*_*e*_ cannot be calculated (Li et al. 2000). Although conventional PK model analysis requires a longer acquisition time than the FPPM method, we believe the utility of *V*_*e*_ in the diagnosis of brain tumors justifies the longer acquisition time of conventional PK model analysis.

Permeability indices, including *K*^*trans*^, have been shown in a number of earlier studies to correlate with glioma grade and to overlap between HGG, metastasis and Lymphoma (Xyda et al. 2012; Awasthi et al. 2012; Zhang et al. 2012; Johnson et al. 2004). Our findings are consistent with these previous studies. Since, the noninvasive estimation of vascular permeability using DCE-perfusion analysis can be useful not only for the diagnosis of the brain tumor (Xyda et al. 2012) but also the malignant potential of the tumor (Patankar et al. 2005), response to biological therapy (Checkley et al. 2003; Keunen et al. 2011), and prognosis (Mills et al. 2006), DCE perfusion imaging with a short acquisition time would contribute greatly to various clinical situations.

Previous studies have revealed that *V*_*e*_ correlates with glioma grade (Awasthi et al. 2012; Zhang et al. 2012), and *V*_*e*_ of lymphoma was higher than that of HGG (Johnson et al. 2004). The present results are in agreement with these previous studies, with *V*_*e*_ showing the highest sensitivity and specificity in the diagnosis of LGG and PCNSL.

The physiological meaning of *V*_*e*_ is still unclear. *V*_*e*_ has been defined as “leakage space” in an initial study (Tofts 1996) and as extravascular extracellular space or its volume in later studies (Tofts et al. 1999). Aref et al. and Aryal et al. reported that interstitial volume fraction measured by DCE-MRI correlated with histologically measured extracellular space fraction in mammary tumor(Aref et al. 2008) and cerebral glioma models (Aryal et al. 2014) in rats. However, Mills et al. and Arlinghaus et al. reported that *V*_*e*_ did not correlate with apparent diffusion coefficient in patients with brain glioblastoma (Mills et al. 2010) and breast cancer (Arlinghaus et al. 2011). Since apparent diffusion coefficient inversely correlates with tumor cellularity (Guo et al. 2002), these results are thought to contradict the correlation between *V*_*e*_ and cellularity.

Additionally, *V*_*e*_ was very high in lymphoma in the present study, which indicates large extravascular extracellular space fraction and low tumor cell density. However, PCNSL is generally a tumor with a high degree of cellularity (Guo et al. 2002). This indicates *V*_*e*_ provides independent information about the tumor microenvironment. Further investigation involving radiological-pathological correlation is needed to reveal the true physiological meaning of *V*_*e*_.

In this study, *V*_*p*_ did not contribute to the diagnosis of the brain tumors, which is somewhat surprising because new blood vessel proliferation in malignant tumors results in increased vascular density. We hypothesized that the *V*_*p*_ of malignant tumors (i.e., HGG, metastasis, and PCNSL) would be higher than LGG, but there was no statistical difference. This preliminary study included a small number of patients and thus statistical power to detect such difference was weak. Further study is needed to determine the meaning of *V*_*p*_ in the diagnosis of brain tumors.

There were other limitations in addition to the small number of patients sampled. We used a DCE-MRI sequence with a short acquisition time and could not compare our results with those from a longer acquisition time. Therefore, we could not evaluate precision of the kinetic parameters themselves. Another limitation was the existence of post-processing software-dependent bias. Kinetic parameters vary between post-processing software (Heye et al. 2013), and our results may have differed if we were to use different software. Finally, the DCE sequence parameters, fixed T1 method, contrast agent, and injection rate also influenced the results. If we were to perform the same experiment with different settings, the results should show the same tendency as in the present study but the kinetic parameters would be different.

In conclusion, we demonstrated the utility of DCE-MRI with a short acquisition time in the differential diagnosis of brain tumors. Operation time was also reduced using automatic vascular function detection and a fixed T1 method. With shortened times for image acquisition, analysis, and operation, the described method shows high feasibility for clinical use.
